# Design and evaluation of the StartingTogether App for home visits in preventive child health care

**DOI:** 10.1186/s12912-018-0310-2

**Published:** 2018-09-15

**Authors:** Olivier Anne Blanson Henkemans, Marjolein Keij, Marc Grootjen, Mascha Kamphuis, Anna Dijkshoorn

**Affiliations:** 1 TNO, Child Health, Schipholweg 77-89, 2316 ZL, Leiden, The Netherlands; 2 Pharos, Arthur van Schendelstraat 620, 3511 MJ Utrecht, The Netherlands; 3 Eaglescience B.V. , Naritaweg 12K, 1043 BZ Amsterdam, The Netherlands; 4 JGZ Zuid-Holland West, Croesinckplein, 24-26 2722 EA Zoetermeer, The Netherlands

**Keywords:** mHealth intervention, Family-centred care approach, Self-management, Parent empowerment, Randomized controlled trial

## Abstract

**Background:**

The StartingTogether program (in Dutch SamenStarten) is a family-centred method for early identification of social-emotional and behavioural problems in young children. Nurses in preventive child health care find it challenging to: determine family issues and need for care; provide education; refer to social services; increase parent empowerment. To mitigate these challenges, we developed and evaluated the StartingTogether App, offering nurses and parents conversational support, tailored education and information on social services.

**Methods:**

A mixed method design, consisting of a qualitative evaluation of the StartingTogether App, with group discussions with nurses (*N* = 14) and a pilot test (*N* = 5), and a randomized controlled trial, evaluating the effectiveness of the app. Nurses (*N* = 33) made home visits to parents (*N* = 194), in teams with or without the app. Nurses were surveyed on the challenges experienced during visits. Parents (*N* = 166) were surveyed on their satisfaction with health care and app. Nurses were interviewed on the benefits and barriers to use the app.

**Results:**

Parents with the StartingTogether App were more satisfied with the visits than parents without (*p* = .002). Parents with a high educational level were more satisfied with the visits than the parents with a low educational level. With the app, their satisfaction level was similar (*p* < .001). Nurses using the app felt more equipped to communicate with parents (*p* = .012) and experienced that parents were more knowledgeable and skilled (*p* = .001). Parents felt that with the app the nurse was more polite (*p* = .02), listened more carefully (*p* = .03), and had more time (*p* = .02). Nurses with the app gave parents more opportunity to ask questions (*p* = .001) and gave clearer answers (*p* < .001). The qualitative evaluation indicated that some nurses needed extra time to develop the habit of using the app.

**Conclusions:**

The StartingTogether App contributes to parents’ satisfaction with home visits. An interaction effect between parents’ educational level and rating of home visits indicated that the app has an additional value for parents with a lower educational level. Applying mobile applications, such as the StartingTogether App, potentially has a positive effect on communication between nurses and parents about the family situation in relation to parent empowerment and the child’s development.

**Trial registration:**

The study is registered with ISRCTN under the number ISRCTN12491485, on August 23, 2018. Retrospectively registered.

## Background

### Dutch preventive child health care

In the Netherlands, approximately 8% of the children experience behavioural and social-emotional difficulties, such as displaying disruptive behaviour, being socially withdrawn, or lacking concentration [1]. These difficulties can be caused by both personal and environmental factors. Children with learning or developmental difficulties, such as speech and language problems, are more at risk to develop behavioural and social-emotional difficulties. In addition, adverse early childhood experiences, such as parental conflict, separation or neglect, can have a negative impact on development and increase the risk of behavioural and social-emotional difficulties [2]. Early identification of and intervention on social-emotional and behavioural difficulties can prevent problems, by optimizing the environment of the child and promoting his or her development [3].

The Dutch preventive child health care (PCH) takes an holistic approach, focusing on both personal and environmental factors. PCH professionals, including doctors and nurses, monitor children’s development during routine assessments at well-child clinics and offer additional interventions at the child and family level [1]. The StartingTogether (ST) program (in Dutch SamenStarten) is a family-centred intervention method for PCH, which aims to contribute to the early identification of social-emotional and behavioural problems in children, aged 0–4 years. The program focuses on building a relationship of trust between the professional and the parent(s), mapping the situation in which the child is growing up, and empowering parents, by expanding their knowledge and improving their skills and self-efficacy [4]. The program is based on the evidence-based Sure Start program in the UK, which offers outreach care (i.e., delivering services in local settings and family environment) to enhance the life chances of young children and their families [5, 6]. Similar to the Sure Start program, ST offers parenting support through home visits and, if necessary, referral to social services in the community. Home visits have been proven to be an effective approach for providing parental support, stimulating children’s development and improving their health [7]. In the Netherlands, this family-centred care approach has proven to contribute to better attuning PCH to the parents’ preferences [8].

The ST program has many components which are described herein. At the well-child clinic, PCH professionals assess the social-emotional and behavioural development of the child and the family situation. The PCH professional uses an interview protocol, called DMO-p (Dienst Maatschappelijke Ontwikkeling [Social Development Services]) [9]. This protocol is based on a bio-ecological model, which emphasizes the importance of understanding bidirectional influences between individuals’ development and their surrounding environmental contexts [10]. The protocol distinguishes five domains: competence of the parent; role of the partner; social support; perceived barriers and life events within the context of the parent (including finance, housing and use of substances); well-being of the child. When parents express a specific need or experience concerns in regard to one or more of the five domains of the DMO-p, the professional provides the parents with educational materials. If more specific support is needed, the professional offers them a home visit by the PHC nurse (in approximately 10% of the cases) [11]. During the home visit, the nurse can personally obtain a clear view of the family situation and has more time for in-depth interviewing with the parent and to build a relationship of trust. The nurse and parents collaboratively re-assess the social-emotional and behavioural development of the child and family situation. Nurses offer parenting support, health advice, and specific support(e.g., post-natal depression). If necessary, the nurse can refer the parents to services in the community, such as a training course on positive parenting.

At the clinic, the nurse adopts a more directive approach, while during the home visits, he or she assumes a more collaborative and/or coaching role. The PCH nurses are trained to apply principles of parent empowerment, as described by Olin and colleagues [12], during these home visits. They strive to recognize, promote and enhance parents’ abilities to meet their own needs, solve their own problems, and activate the necessary resources in their community in order to feel in control of their own lives [13]. They take a positive approach, activate parents as change agents in meeting their children’s physical and mental health needs, provide structure (e.g., goal, clarity, timeliness), and discuss important relational aspects (i.e., reliability, openness, equality, collaboration and attending to needs and possibilities).

Home visit play a key role in the ST program for outreaching and parent empowering child health care. However, previous evaluations of the program in the Netherlands showed that nurses experience a number of challenges during these visits [9], namely:

1. Identifying the family situation and parents’ associated care needs can be arduous. Parents have difficulties verbalizing their needs. Also they are overwhelmed by a multiplicity of family issues and lack knowledge of how to resolve these issues;
2. Nurses lack materials, such as a guide for conversational techniques, to provide information and communication tailored to the family situation and care need;
3. Nurses do not always have the interventions at their disposal to apply during the home visit;
4. Parents are not always referred to relevant social services. An overview of the available services in the community is missing.

These challenges obstruct the identification of personal and environmental factors, influencing the children’s social-emotional and behavioural development. Also, they hinder the empowerment of parents and their ability to cope with their family situation independently.

### Apps for preventive child health care

Mobile eHealth applications, also called mHealth apps, are increasingly used to supplement health care interventions. Benefits of apps are that they are mobile and can be easily applied during home visits. For instance, professionals can use mobile devices to interact with their clients to access and share online information, or for assessment purposes. Recent meta-analyses have shown that mobile technologies in behavioural interventions lead to better treatment outcomes than interventions without any form of mobile technology [14]. Also, they can contribute to the effectiveness of health care delivery services [15] and enhance the efficacy of health care interventions, for example by increasing adherence to guidelines, enhancing health surveillance, reducing medication errors or decreasing rates of redundant of inappropriate care [16].

Table 1 lists various mHealth apps (Smartphone or Tablet PC) that are developed to support PCH. These apps offer support for monitoring children’s development, parent education and empowerment or support for PCH professionals, such as social workers. However, none of these apps cover all steps of a home visit, nor do they offer communication support for the conversation between the nurse and parents. Moreover, none of the apps has been evaluated on its effectiveness in regard to identifying the family situation, determining care needs and increasing parent empowerment.

**Table 1:** mHealth apps for preventive child health care

Function	App (country)	Goal
Monitoring child development	*My child’s eHealth* record (Australia)	Child’s health record with information about the child’s development
	*Baby Connect, Baby Food Pee Poo, and Total Baby* (US)	Graphical reports and charts, weekly averages, medicine, vaccine and growth tracking, and allergies. Also, timers, notifications, reminder alarms, and appointments for doctor visits
Parent education and empowerment	*WhatToExpect* (US)	Day-by-day pregnancy guide, with personalized content, parenting news and health information. Can be connected to a community of expecting moms
	*Breastfeeding Management* application (US)	Information about breastfeeding, such as guidelines for the use of medications during breastfeeding
Support for child health care professionals, such as social workers	*Child Development 0–6 Years* app (Ireland)	Information on child developmental norms relevant to the 0–6 year’s age group

To mitigate the challenges experienced during the ST home visits, the StartingTogether App (ST App) was developed. It runs on a Tablet PC, which the nurse uses during the home visits, in collaboration with the parent. It covers: 1) textual and visual conversational support for nurses and parents, 2) education and information on social services in the community, tailored to the care need and the living location, 3) email reports of the home visit for both the parents and the nurse and 4) tools for the nurse to prepare the home visit. See section Intervention for further details.

The aim of this study is twofold: 1) to describe the development process of the ST App and 2) to evaluate its effectiveness in a) facilitating a partnership between professionals and parents b) enhancing the identification of the family’s needs c) empowering parents to address these factors themselves and effectively cope with their family situation.

## Methods

### Evaluation framework

**Fig. 1:**
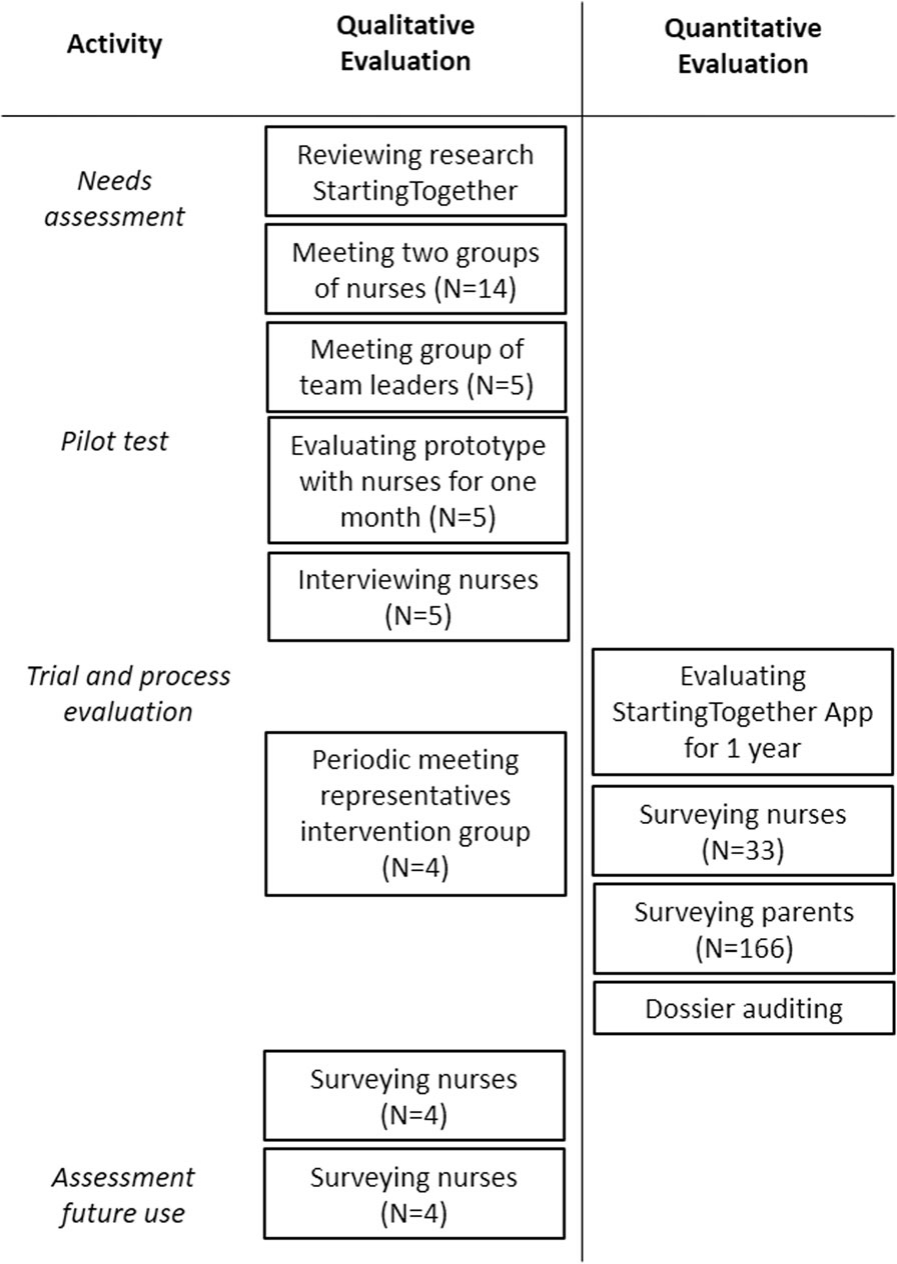
Flow chart of quantitative and qualitative evaluation

To fulfil our research aim, we used an exploratory sequential mixed method design, in which the qualitative study is combined with the quantitative [17]. The value of this design exists in the fact that results are enhanced to a greater level than the quantitative or qualitative component on their own, and that the approach allows for development and evaluation of the app at the same time (see Fig. 1). The first part of the study consisted of a qualitative, formative evaluation of the ST App, with the aim of improving its design and implementation. The second part of the study consisted of a Randomized Controlled Trial (RCT) to evaluate the effectiveness of the ST App when applied during home visits. Finally, we have integrated the results of the two parts, to come to strengths and weaknesses of the app in regard to future use, and strategies for implementation.

### Qualitative study design

The main research questions for the qualitative study, that guided the evaluation were: What needs do nurses and parents have, with regard to the home visits? How can the ST App be applied during home visits, so as to contribute to patient-centred quality of care? What issues are important for further implementation? To answer these questions, we conducted: a needs assessment; a pilot test; a process evaluation during the iterative development of the app; and an assessment for future use.

The study design was based on a user centred design (UCD) approach for mobile applications, situated cognitive engineering, and intervention mapping. UCD for mobile applications is a process in which the needs, wants, and limitations of end users of mobile apps are given extensive attention at each stage of the design process [18]. Situated cognitive engineering is an iterative development process with active involvement of users [19]. This process stems from UCD, but has an additional scientific research component. It aims to establish and test theories from the domain for which the application is developed. Finally, intervention mapping is a development process of health promotion programmes. It aids mapping the path from recognition of a health need or problem to the identification of a solution [20].

### Recruitment of participants

The participants were nurses and team leaders working at the PCH Service in Amsterdam. A total of 13 PCH team leaders and approximately 35 nurses were invited to participate in focus groups for the needs assessment, of which five team leaders and fourteen nurses volunteered.

### Qualitative data collection

For the needs assessment, two focus groups with the 14 nurses and one focus group with the five team leaders of the PCH service were held in Amsterdam. During these group meetings, they shared their experiences with home visits and expressed how they felt a mobile application could provide them the necessary support. The following topics, as derived from previous research on StartingTogether [9], were used to stimulate the discussion: preparation for the home visits; identifying family needs; strengthening parents skills and motivation; referral to services; offering additional care; other points in regard to StartingTogether. Also during the focus groups, a first mock-up of the app was shown to the nurses for inspiration. It consisted of rough sketches of its interface for navigation and possible functionalities (i.e., media functionality and referral functionality). They were invited to reflect on this first mock-up, provide additional suggestions and to give suggestions for improvement.

Based on the challenges elicited from previous research [9] and the needs assessment, a first version of the ST App was build. It was pre-tested with nurses (*n* = 5) from the intervention group, divided across different teams. They used the app for one month in a pilot field-test and we evaluated their experience through short interviews. The outcomes of the interviews were used to refine the app. We added more “affordance” to improve the usability of the app, such as buttons for navigations in addition to tabs at the bottom of the screen, redesigned the emoticons as to make them more recognizable, added a direct search option for educational materials (without selecting topics of discussion) and used simpler wording to make the app easier to understand for a layperson. Finally, we resolved some final technical bugs. A process evaluation took place parallel to the RCT (see part Quantitative study design), to ensure that the ST App matched with the PCH work process and needs. Focus groups with representatives of the different intervention group teams (*N* = 4) were held to investigate the professionals views about the app. They were asked about their experiences with the app and suggestions for possible improvements for the future. After the RCT, we administered a survey among the nurses in the intervention group. We asked them about strengths and weaknesses of the app in regard to future use.

### Quantitative study design

The second part of the study consisted of a Randomized Controlled Trial (RCT) to evaluate the effectiveness of the ST App when applied during home visits. The main research questions for the quantitative study to measure the effectiveness were: how satisfied are nurses’ and parents’ with the home visit; how do they rate the usability of the app; and what is the proportion of valid referrals made after the home visit? Secondary outcome measures included demographics of the nurses, children and parents, to explore potential interaction effects (see Table 2).

**Table 2:** Overview of primary and secondary outcome measurements per group

Outcome measures	Nurses	Parents
Primary outcome measures	Time of measurement	
Evaluation of home visit: challenges experienced during the Starting Together	At the end of the visit	–
Evaluation of home visit: patient-centred health service, quality of care, overall satisfaction	–	At the end of the visit
Usability of StartingTogether App	–	At the end of the visit (intervention group)
Secondary outcome measures	Time of measurement	
Demographics nurse	Onset of study	–
Demographics child	At the end of the visit	–
Demographic parents	–	At the end of the visit

### Recruitment of participants

The participants were PHC nurses in Amsterdam. They made home visits to parents with children aged 0–4 years in which family issues and/or needs for support were identified with the DMO-p. A total of 17 PCH teams (120 nurses) were invited to participate in the study, of which 9 teams volunteered (*N* = 34 nurses). Reasons not to participate included lack of time or capacity to participate in the study. There were no exclusion criteria. All PCH teams in Amsterdam were invited to participate in the study and all parents and nurses who had agreed to participate were included in the study. One nurse dropped out early in the study and her data were excluded from the analysis.

### Intervention

**Fig. 2:**
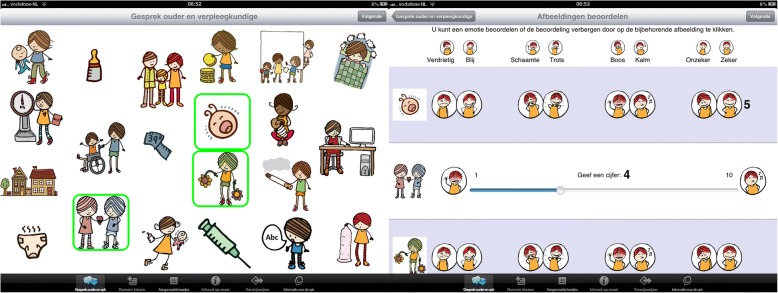
ST app: Conversational support

Figure 2 shows the first function of the ST App: textual and visual conversational support for nurses and parents. The parents first select one or more pictograms (e.g., sleeping child or a couple with a heart) that illustrate the topic(s) they want to discuss with the nurse, eliciting a conversation about their family situation (e.g., the babies’ sleeping rhythm, or the relationship with their partner). Then, they value these issues with the use of emoticons (i.e., sad/happy, angry/calm, insecure/secure, and shameful/proud). Parents can rate their current emotional state on a scale from 1 through 10 (e.g., 4, somewhat sad), define their goal state (e.g., 5 or 6, somewhat happy) and determine their personal needs. These ratings are meant to promote self-reflection, prioritization of their needs and self-activation. Also, they can determine what they can do themselves, with the help of their social environment, to achieve this goal state.

The concept of selecting and discussing pictograms is derived from *context mapping.* This technique consists of people selecting and discussing artefacts (in this case pictograms) and to make their tacit experiences and feelings explicit [21]. This approach can aid parents and nurses to understand the bidirectional influences between their contexts and their child’s development. Also, it can aid them to talk about sensitive topics, which is known to be a barrier for nurse-parent interaction [22]. The valuing of the pictograms stems from both context mapping and solution focused (brief) therapy (SFBT). In SFBT, through precisely constructed questions about the family situation, parents can focus on identifying their goals and generating a detailed description of what life will be like when the goal is accomplished [23]. Thus, the focus lies on constructing situations in which the problem is resolved rather than solving problems.

**Fig. 3:**
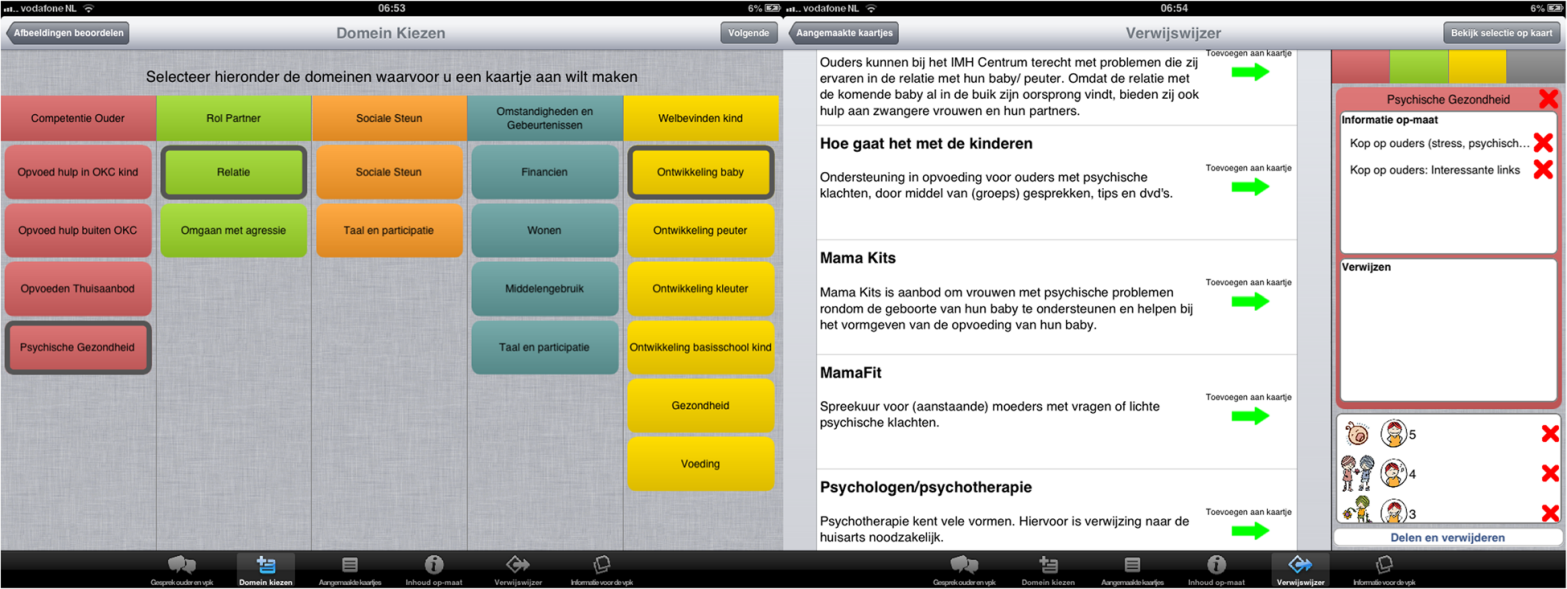
ST app: Tailored education (websites, flyers, videos) and information on social services

As illustrated in Fig. 3, the app provides educational materials (i.e. websites, flyers and videos), and lists social services in the community, tailored to the family’s situation, need and living location. Examples are an educational video on shaken baby syndrome, pamphlets on upbringing and websites with self-management programs for parents with psychological problems. The educational materials and social services in the community were provided and reviewed by the nurses and team leaders, and added to the app by the researcher, before and during the study. The DMO-p domains are used to guide the selection of relevant materials, tailored to the social-emotional and behavioural development of the child. The nurse selects one or more domains, based on the parents’ selection of topics and evaluation of their family situation. Earlier research has shown that tailoring can enhance the users’ participation and engagement in the intervention and, in turn, patient empowerment (i.e., knowledge and skills for self-management) [24]. After choosing one or more domains, the app provides relevant education and social services in the community. The parents and nurse can browse through these services and compare them; who is the target group, what is the service, how much does it cost and is it in the neighbourhood? The locations of the services in the community are displayed on a map, showing the current (home) location and the locations of the selected service. This is meant to facilitate shared decision-making about which care will be provided, which can contribute to parent empowerment, as well as to the parents’ satisfaction in regard to the home visit [25].

The app keeps a record of the home visit, which is sent by email to the parent at the end of the visit. The report covers the family’s situation and evaluation, the selected educational materials and information about social services (with contact details), notes and follow-up appointments. With the parent’s consent, the nurse can send the email to him- or herself, for future reference. For privacy purposes, once the email is sent, the record is removed from the app. Finally, the app offers tools for the nurse, such as official websites for PCH-professionals on youth and upbringing. Here, they can access the relevant care standards for preparation of the home visit.

### Quantitative data collection

**Fig. 4:**
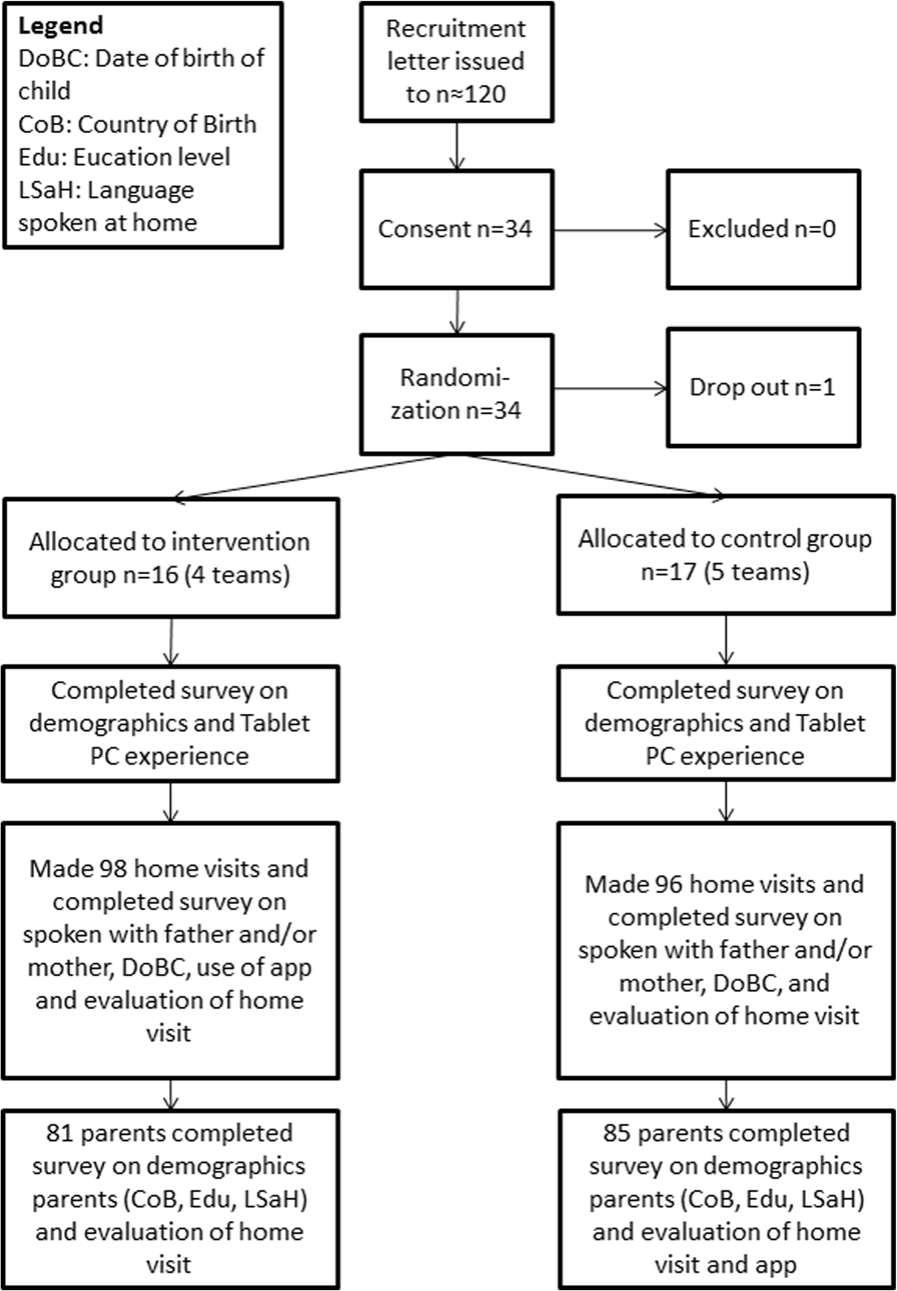
Flowchart of RCT

The RCT was conducted over a period of one year (October 2012 through September 2013). As illustrated in Fig. 4, teams of nurses were assigned to the intervention group (STA), which made home visits with the ST App, or the control group, which made home visits as usual (CAU). We applied a stratified randomization method: four teams were allocated to the intervention group (*N* = 16) and five teams were allocated to the control group (*N* = 17). All participating nurses received a two-hour training to complete the survey and administer it to the parents. The STA nurses received a four-hour training to use the ST App, covering a theoretical and practical module.

The nurses in the STA condition made 98 home visits and the nurses in the CAU made 96 home visits. After each home visit, nurses completed a survey about the home visit and invited parents to also complete a survey, after informed consent. The survey for the parents was in four languages: Dutch, English, Arabic and Turkish. The STA group completed the survey on the Tablet PC (a special survey app was developed) and the CAU group completed the survey on paper.

The survey for parents contained questions about their demographics (country of birth, language spoken at home, educational level, age of child). Subsequently, it asked to evaluate the home visit with the use of the validated (α = .75) Consumer Quality Index for PHC (CQ-Index JGZ), based on the American Consumer Assessment of Healthcare Providers and System [26]. The CQ-Index JGZ assesses patients’ views, which are essential to provide a patient-centred health service and to evaluate the quality of care [27]. Table 3 lists the items proposed to the parents to rate on a 5 point scale (1, not agree at all, through 5, fully agree). Finally, parents were asked to rate their overall satisfaction with the home visit on a scale from 1 (lowest) to 10 (highest). Parents in the intervention group were also asked to rate the app’s usability on a scale from 1 (lowest) through 5 (highest).

**Table 3:** Parents’ evaluation of the home visits, with and without the StartingTogether App, controlled for controlled for the characteristics of the family (covariates) (*N* = 166)

Covariate	Dependent Variable	df	Mean Square	F	Sig.
Condition (with or without app)	The nurse understood what the parents wanted to talk about	1	1.12	2.23	.14
	The nurse was polite to the parent	1	2.35	5.05	.03
	The nurse listened carefully to the parents	1	2.44	5.45	.02
	The nurse had enough time	1	3.54	5.23	.03
	The parents could ask questions	1	3.66	13.03	.001
	The nurse provided clear answers	1	4.00	9.38	.003
	The advices were usable for the parent	1	.01	.01	.92
	The parents were well referred (if relevant)	1	.08	1.00	.76
	The rating of the parent of the home visit	1	15.02	10.60	.002
Education level parent (high/low)	The nurse understood what the parents wanted to talk about	1	6.32	12.60	.001
	The nurse was polite to the parent	1	4.65	10.00	.002
	The nurse listened carefully to the parents	1	4.57	10.20	.002
	The nurse had enough time	1	3.94	5.82	.02
	The parents could ask questions	1	3.65	12.99	.001
	The nurse provided clear answers	1	.74	1.74	.19
	The advices were usable for the parent	1	.10	.13	.72
	The parents were well referred (if relevant)	1	9.73	11.40	.001
	The rating of the parent of the home visit	1	.82	.58	.45

The survey for the nurses contained questions about their experience with Tablet PCs and apps, at work and at home, the characteristics of the child (gender and aged), and which functions of the app they used. In addition, it asked to evaluate the home visit, in relation to the challenges experienced during the ST home visits. Table 4 lists the items proposed to the nurses to rate on a 5 point scale (1, not agree at all, through 5, fully agree).

**Table 4:** Nurses’ evaluation of the home visits, with and without the StartingTogether App (*N* = 33)

Item	With StartingTogether App		Without StartingTogether App		*P*-value
	Mean	SD	Mean	SD	
The care need was clear for the nurse	28	.71	38	.64	.32
The care need was clear for the parent	41	.72	30	.79	.31
The parents and nurse had a shared view of the care need	06	.61	05	.77	.91
The nurse was capable of communicating with the parent(s)	30	.46	01	.79	.002
The nurse was capable of informing the parent(s)	25	.52	15	.79	.32
The parents knew how to cope with their family issues at the end of the home visit	28	.82	3.81	.81	.000
The parents felt competent to cope with their family issues at the end of the home visit	3.97	.98	3.53	.84	.001
The parents were motivated to cope with their family issues at the end of the home visit	24	.69	17	.85	.56
The parents had the intention to follow the referral advice at the end of the home visit (if relevant)	30	.70	39	.70	.56

As part of their care as usual, nurses in both groups made reports of home visits in the electronic child dossier of the PCH Service in Amsterdam. A dossier audit was conducted to determine the number of valid referrals for each group during the RCT.

### Data analysis

For the qualitative study, the data was analysed using a content analysis approach. The conversations during the focus groups and interviews were recorded, and moderators made notes during the meetings. The principles of interpretive description were used to guide the data collection and analysis of the needs assessment, pilot field-test, and the interviews about the process and implementation.

For the quantitative study a power calculation was run. The use of the ST App was used as the dependent variable and the quality of home visits as rated by the parents as the primary independent variable. We applied a formula for statistical superiority design to calculate the a sample size, assuming that our new treatment ST App is more effective than a standard treatment (CAU) from a statistical point of view (Lesaffre, 2008). The main hypothesis was that the use of the app would lead to a higher rating for quality of care perceived by the parent. Other hypotheses were the use of the app would lead to better establishing parents’ care need, offering tailored advice and referral, and parent empowerment. Power calculation showed that a sample sizes of 15 nurses in group one and 15 nurses group two (assuming 20% drop-out), each including 10 home visits with parents, would achieve 71% power to detect a difference between the group proportions of 0.100. The group one proportion was assumed to be 0.250 under the null hypothesis and 0.350 under the alternative hypothesis. The test statistic used is the two-sided Z test (pooled). The significance level of the test was 0.05.

Data of the quantitative evaluation were checked for normal distribution using graphical summary of data, assessment of skewness, descriptive statistics, and tests of normality. A comparison was made between the STA group and CAU group in regard to the evaluation of the home visit by nurse with a non-parametric Two Independent Samples test (Mann Whitney). A comparison was also made between the STA group and CAU group in regard to the evaluation of the PCH by parent with a multivariate analysis. We analysed the effect of the app on the different dependent variables reported by the parents, controlled for characteristics of the family: child’s age, child’s gender, parent(s) spoken to during the visit, country of birth parent, language spoken at home, and education level parent. Finally, the number of valid and invalid referrals was compared with an Chi square-test.

From the dossier audit reports, we measured the number of referrals per group and validity of the referral. The auditor, a staff nurse at this Service, retrieved reports from the period November 2012 through August 2013. She reviewed if the parent was referred to social services in the community or not. Then, she reviewed if the (non)referral was valid. Validity of referrals was determined through level of adherence to PCH evidence-based protocol [28] The auditor looked at the topic (e.g., sleep), type of issue (e.g., child has difficulty sleeping), the level of complication (e.g., past week or multiple months), and the prescribed intervention or referral (e.g., sleep training or referral to psychologist). Referral was valid or invalid based on how accurate the nurse followed the protocol. For reliability of the audit, at the beginning of the review, a second auditor reviewed 10% of these reports, and the two auditors discussed the reviews which differed to come to a consensus. The goal of the discussion was to increase the main auditor’s rating accuracy. By discussing if and when a dossiers was valid or not, based on the PCH protocols and expertise of two auditors, the main auditor could sharpen her rating skills. The inter-rater reliability was 0.79 (Cronbach’s alpha).

### Ethics, consent and permissions

At the onset of the RCT, parents, nurses and their team coordinators received a letter with information about the study (goal, results, data processing and rights) and an invitation to participate. The nurses, parents and team coordinators, who were willing to participate in the study, gave their informed written consent. The study protocol was approved by the ethics committee of TNO (Nederlandse Organisatie voor Toegepast Natuurwetenschappelijk Onderzoek [Netherlands Organisation for Applied Scientific Research]) registered under the number 05101117.

The digital surveys were directly emailed from the Tablet PC to the TNO main researcher. The paper surveys were put in sealed envelopes by both parents and nurses. Thus, the surveys completed by the parents were collected without the nurses reviewing them. These envelopes were stored in a dedicated locker at the well-child clinic and periodically retrieved by the TNO researcher (time between retrievals was a maximum of 3 weeks). After collecting the data from the surveys, the envelopes were stored in a TNO archive, only accessible by the researchers of TNO involved in the project. The data were saved in a digital safe on the TNO server, only accessible for the involved researchers of TNO. Data and envelopes were stored for a 10 year period, after which they will be destroyed. The audit complied with the privacy law and administration of patient data. Audit data could only be retrieved anonymized and could therefore not be matched with the survey data.

## Results

### Qualitative findings

#### Participants

Five team leaders and 14 nurses participated in the needs assessment. The nurses came from different regions in Amsterdam, namely the Centre (*N* = 3), West (*N* = 2), North (*N* = 3), Amstelland (*N* = 1), East (*N* = 3) and New-West (*N* = 2). The residents of these regions vary in ethnicity, Socio-economic status (SES) and family issues.

#### Outcomes needs assessment

As listed in Table 5, four important topics were addressed in the needs assessments by the nurses, which were: preparation for the home visits; referral to services; identifying family needs; strengthening parents skills and motivation. For these topics, three needs were stated, each with their own rationale. First, nurses indicated that it would be helpful to have a map of social services in the community and that each service listed would present information for the parents, such as registration procedures, waiting lists, costs and location. Second, they asked for additional instruments supporting the conversation with parents to assist in identifying and articulating root causes of sensitive family issues. Third, these instruments should be adaptive, to match the family’s profile (e.g., intrinsic/extrinsic motivation, level of empowerment and autonomy), in order to achieve shared decision making.

**Table 5:** Outcomes of the needs assessment

Topic	Needs elicited	Rationale
Preparation for the home visits	A map with social services in the community within reach of the family, listing information for the parents, such as registration procedures, waiting lists, costs and location	An overview of available services in the neighbourhood is lacking. Also, these services change frequently. This can make nurses feel unprepared for the home visit, especially if they have to consult the office to discuss the next steps and potential referral to other social services
Referral to services		
Identifying family needs	Instruments, in addition to the standard DMO-p, to identify sensitive issues causing the family needs	Time is needed to build a relationship of trust and make the parents feel comfortable to discuss sensitive topics. In some cases, families have multiple problems at the same time, and the nurse has to help ordering and prioritizing these problems to know where to start.
Strengthening parents skills and motivation	The communication should be adaptive and fit the family’s profile, in order to achieve shared decision making	There is variation in families’ request and need for support, due to differences in intrinsic/extrinsic motivation, level of empowerment and autonomy

When asked about suggestions for the ST App, nurses indicated they wanted it to contain documents they generally use. Team coordinators suggested a systematic approach in the app, as it is a requirement for evidenced-based work. That is to say, an approach based on scientific methods and applied similarly by the different nurses. Furthermore, the nurses requested that the functions of and interaction with the app matched their standard work methods in preparation for and during home visits.

The responses to the mock-up varied. First, they were shown a draft of a “media functionality” of the app, which contained information and educational media (fact sheets, video, websites) to aid communication with the parents about their care question(s). Nurses were positive about this component and felt that the app contained media they generally use, and that the form and functions of the app matched their standard work methods. A suggestion for improvement, was tailoring the media more to the parents than to the nurses, so they can walk through the information in the app together, without difficult language or jargon. Another suggestion was to offer the materials to the parents online, so they can look things up themselves after the home visit.

The “referral functionality” in the app contains a map of social services in the community. Nurses positively evaluated this functionality and found it helpful to find relevant information about available services. As suggestions for improvement, they indicated it could be useful to have an email function, so that when parents are being referred, they could send an email to the concerned institution together with the nurse. Also, they suggested a function that allows parents to evaluate the social service. Team coordinators recognized a systematic approach in the app, but provided a number of suggestions for improvement in regard to the interaction (for example, legend for the emoticons).

#### Outcomes pilot field-test, process evaluation and assessment future use

The outcomes of the needs assessment and suggestions for improvement of the draft version were used to build a first version of the ST App. Subsequently, the app was piloted for one month in a field-test with nurses (*n* = 5) from the intervention group. We evaluated their experience through short interviews at the end of the pilot. In general, the nurses and coordinators expressed that the app fulfilled their needs and followed their suggestions formulated in the group meetings. Suggestions for improvements, which were mainly at the level of the interface (e.g., navigation through use of buttons at tabs, reposition of items in the screen), were applied in the ST App evaluated in the RCT.

During the process evaluation, nurses mentioned several benefits of the ST App. They felt that the app provided parents insight into their family situation and care needs; they felt more able to relate to the parents (during the home visit, they could literally sit next to the parents with the Tablet PC on their lap or on the table); that the textual and visual conversational support helped parents to express their needs; and they could collaboratively set up a personal plan to address the family situation. The nurses also suggested a number of challenges for the ST App for future use. To implement the app on a broader scale, nurses have to be willing to use the app for the first time. They might need to experience the benefits of the app themselves and need time to adopt the app, in order to develop the habit of using the Tablet PC and the app during home visits. Also, it is important that the management expresses its support for the app throughout the organisation. Finally, nurses missed a tool to easily maintain the educational materials and information on the app themselves (during the study, this was done by the experiment leader). The results of process evaluation are used to further improve the app and develop a training of the app.

### Quantitative findings

#### Participants

PHC nurses from nine teams working in Amsterdam participated in the RCT. In the STA group, nurses from four different teams (*N* = 16) visited 98 families of which 85 parents agreed to complete a survey. In the CAU group, nurses from five teams (*N* = 17) visited 96 families, of which 81 parents agreed to complete a survey. One nurse allocated to the control group dropped-out and her data were excluded. The participating nurses were all women. The average age of the nurses in the intervention group was 43.8 years (SD = 13.1) and in the control group 37.1 years (SD = 10.2) (*p* = .11). All nurses had a Bachelor degree in nursing. More than half of the nurses in the intervention and the control group (respectively 62.5% and 52.9%) had no previous experience with a Tablet PC.

As listed in Table 6, the nurses visited parents with different countries of birth (such as, the Netherlands, Morocco and Turkey) and educational levels (raging from none to Master degree). Parents spoke different languages at home (such as, Dutch, Turkish and Arabic). The average age of the visited children in the control and the intervention group was, respectively, 4 and 6 months. The country of birth, language spoken at home, educational level and age of the child did not differ significantly between the groups.

**Table 6:** Demographics of parents (*N* = 166)

Item	With StartingTogehter App	Without StartingTogehter App	Total
Country of birth			
Netherlands	45	41	86
Morocco	9	16	25
Turkey	7	4	11
Other	25	19	44
Total	86	80	166
Language spoken at home			
Dutch	45	49	94
Turkish	11	6	17
Arabic	8	8	16
Other	22	17	39
Total	86	80	166
Education level			
None	2	2	4
Low (Primary)	8	6	14
Average (General Secondary Education)	21	26	47
High (BA, MA)	55	46	101
Total	86	80	166

Parents and nurses discussed the following topics derived from the five domains of the DMO-p. In approximately half of the home visits, the parents and nurses spoke about parenting (59.3%) and the well-being of the child (48.5%). In approximately one in six home visits they spoke about the psychological well-being of the parent (18.0%) and nutrition of the child (including breastfeeding) (15.5%). Other topics discussed less frequently were the relationship between parents, aggression at home, language, finance, housing, use of substances, and disability of the child.

#### Ratings for the home visits

Table 3 lists how the parents rated the home visit with and without the ST App, along the CQ-Index JGZ items, controlled for the characteristics of the family (covariates): age child, gender child, parent(s) spoken to during the visit, country of birth parent, language spoken at home, education level parent. In addition to the use of the app, education level of the parent affect the evaluation of the home visit, see section below ‘Interaction effects: Educational level and satisfaction ratings’. The other covariates did not significantly affect parents’ evaluation of the home visits.

Some items received a significantly higher rating with the app than without the app. The CAU group (without app) gave the visits an overall rating of 8.04 (SD = 1.03), on ascale from 1 (lowest) through 10 (highest). The STA group gave an average rating of 8.82 (SD = 1.21) (F(1) = 10.60, *p* = .002). In regard to the nurse being polite, STA parents gave an average rating of 4.58 (SD = .75) and CAU parents an average rating of 4.46 (SD = .60) (F(1) = 5.45, *p* = .02). In regard to listening carefully by the nurse, STA parents gave an average rating of 4.68 (SD = .71) and CAU parents an average rating of 4.43 (SD = .61) (F(1) = 5.05, *p* = .03). In regard to the nurse having enough time, STA parents gave an average rating of 4.61 (SD = .67) and CAU parents an average rating of 4.41 (SD = .76) (F(1) = 5.45, *p* = .02). In regard to the opportunity to asking questions, STA parents gave an average rating of 4.64 (SD = .51) and CAU parents an average rating of 4.28 (SD = .66) (F(1) = 13.03, *p* = .001). In regard to the clarity of the answers provided by the nurse, STA parents gave an average rating of 4.56 (SD = .59) and CAU parents an average rating of 4.20 (SD = .66) (F(1) = 9.38, *p* = .003). The multivariate analyses also showed that education level explained variance in how parents rated how the nurse’s understanding of what the parents wanted to talk about (F(1) = 12.60, *p* = .001) and the quality of their referral (if relevant) (F(1) = 11.40, *p* = .001), whereas the use of the app did not (F(1) = 2.23, *p* = .14; (F(1) = 1.00, *p* = .76).

Table 4 lists how the nurses evaluated the home visit with and without the ST App, regarding the challenges experienced during home visits. Also according to the nurses, some items received a significantly different rating with and without the app. In regard to communicating with the parent, STA nurses gave on average a significantly higher rating (M = 4.30, SD = .46) then CAU nurses(M = 4.01,SD = .79) (Z = 2.66, *p* = .008). In regard to the parent’s knowledge how to address the family situation, STA nurses gave on average a significantly higher rating (M = 4.28, SD = .82) than CAU nurses (M = 3.81, SD = .81) (Z = 3.97, *p* < .001). In regard to the parent’s skills to address the family situation, STA nurses gave on average a significantly higher rating (3.97, SD = .98) than CAU nurses (M = 3.53, SD = .84) (Z = 3.35, *p* = .001).

During the audit, 93 reports from the intervention group and 95 reports from the control group were reviewed. In the intervention group, 33% of parents were referred, while in the control group, 50% of the parents were referred, which is a significantly higher percentage (X^2^(3) = 55.26, *p* < .001). The auditor rated 96% of the (non)referrals in the intervention group as valid and 95% in the control group.

#### Interaction effects: Educational level and rating of home visits

**Fig. 5:**
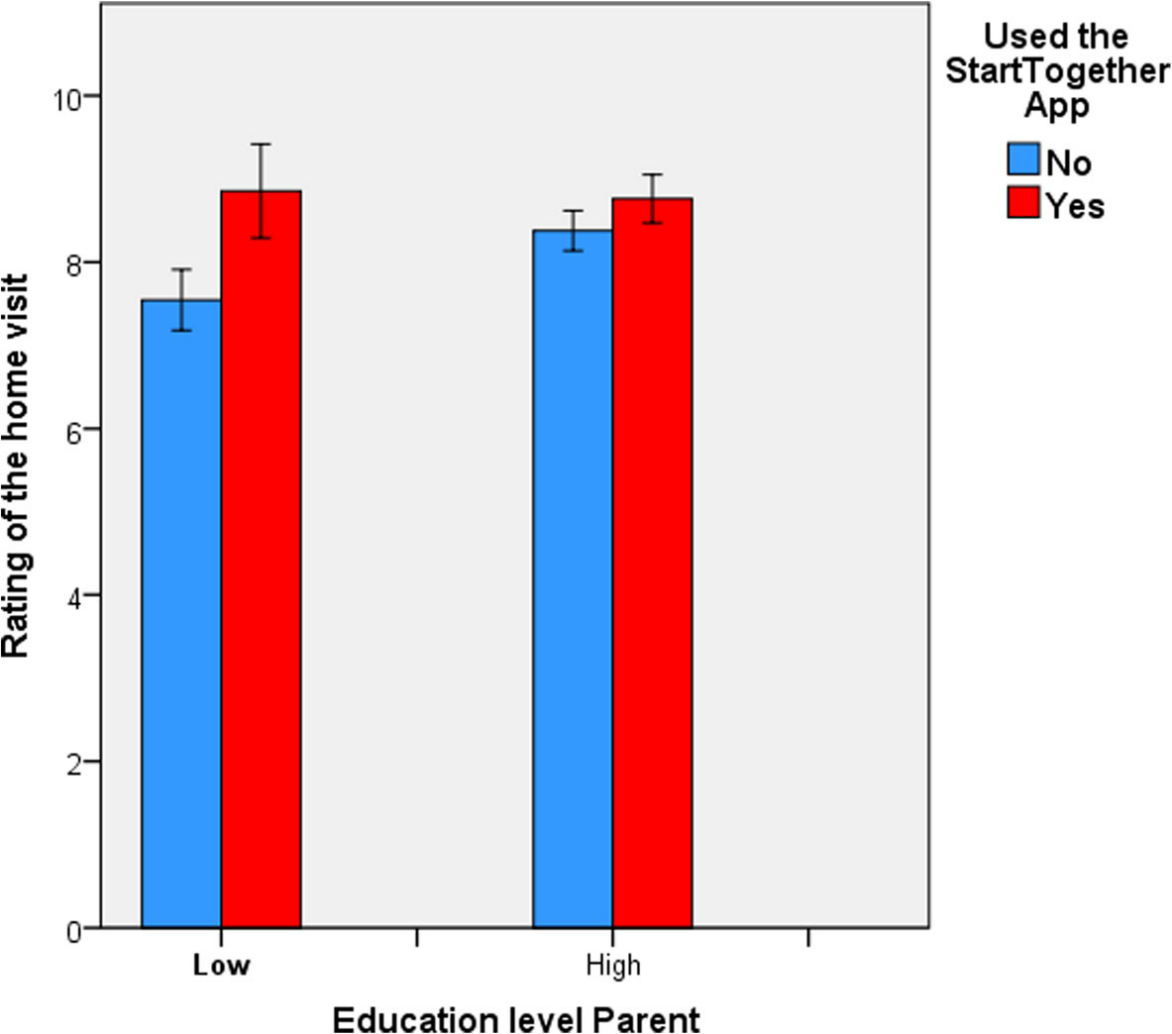
Evaluation of home visit by parents with high and low educational level with and without StartingTogether App

As listed in Table 7, a linear regression, with age child, gender child, parent(s) spoken to during the visit, country of birth parent, language spoken at home and education level parent as predictors showed that variance in parents’ rating of the home visits was explained by educational level (Beta = .16) (R^2^ = 03, *p* < .05). When we divided parents along the median in two educational level groups (high and low), data showed that parents with a high educational level gave the home visits an average rating of 8.58 (SD = .97), while parents with a low educational level gave the home visits an average rating of 8.13 (SD = 1.41) (F(162) = 2.46, *p* = .015). As illustrated in Fig. 5, parents in the CAU group with a high educational level gave home visits an average rating of 8.37 (SD = .78) and parents with a low educational level gave the home visit an average rating of 7.47 (SD = .99). In the STA group, parents with a high educational level gave home visits an average rating of 8.76 (SD = 1.07) and parents with a low educational level gave the home visit an average rating of 8.90 (SD = 1.45). The analysis showed an interaction effect (F(57) = 4.91, *p* < .001).

**Table 7:** Model for variance in parents’ rating of the home visits, explained by parent and child characteristics

Coefficients Parents’ rating of home visit						
Model	Variable	Unstandar-dized B	Coefficients	Standardized Coefficients Beta	t	Sig.
1	(Constant)	7.76	.33		23.19	.000
	Education level	.14	.07	.16	1.98	.049
Excluded Variables						
Model	Variable	Beta In	Partial Correlation	Collinearity Statistics Tolerance	t	Sig.
1	Age child	−.06	−.06	1.00	−.78	44
	Country of birth	.06	.06	.96	.78	.44
	Language spoken at home	.99	.09	.93	1.07	.29

#### Usability ratings

On a scale from 1 (lowest) through 5 (highest), parents gave the ST App an average rating of 4.21 (SD = .81). The nurses used different functions of the ST App during the home visits: 58.2% used the textual and visual conversational support for nurses and parents; 80.6% provided education (website, flyer, video) and/or information on social services in the community; all nurses sent an email report of the home visit for the parents and the nurse; 24.5% used the tools for the nurse, such as professional websites on youth and upbringing.

## Discussion

The aim of this study was to describe the development process of the ST App and to evaluate its effectiveness in improving the quality of care of PCH home visits. The first part of the study consisted of a qualitative evaluation. Nurses were positive about the app. They felt that it contained media (fact sheets, videos and websites) they generally use; the form and functions of the app matched their standard work methods; it provided parents with insight in their family situation; helped parents to express their needs; and enabled nurses to better connect to the parents and address their situation. Lastly, the referral aid turned out to be helpful in finding information about available services, and team coordinators were positive about the systematic approach in the app.

The second part of the study consisted of a quantitative evaluation of the PHC with and without the ST App. The ST App had additional value for the ST home visits regarding communication and parent empowerment. Overall, parents gave the home visits a positive rating. However, when the ST App was used, their ratings improved. Parents felt that the nurse with the app listened more carefully. Also, they felt the nurse had more time and gave them more opportunities to ask questions and provided clearer answers. Finally, parents felt that with the app nurses were more polite. This is notable as previous research found that mobile applications can disconnect us from the surrounding social environment (e.g., nurses have less attention for the parent as they are more focused on the app) [29].

The nurses in the STA group felt that the communication with the parents improved. Also, parents were more knowledgeable and skilled to address their family situation independently, as to contribute to their child’s development. These findings are in line with the findings of previous research, that has shown that the use of mobile technology in health care interventions can improve communication between clients and health care professionals [30]. With the ST App, parents were less often referred; however, in both groups most referrals were rated as valid. This is in line with previous research, that has shown that the use of mobile technology in (preventive) health care interventions can decrease rates of potentially redundant or inappropriate care [6]. Finally, parents with a low educational level rated the home visits lower than the parents with a high educational level. However, when the app was applied, the ratings were similar for both groups. A possible explanation for this interaction effect is that parents with a low educational level have more difficulty expressing their needs, and therefore benefit more from the textual and visual support than parents with a high educational level. Another explanation is that parents with a high educational level find it easier to look up information and ask for help themselves, without the help of the nurse, and benefit less from the information and educational materials provided by the app. Illustratively, we found that education level explained variance in referral. Parents with a higher education level were more likely to be referred accurately to local social services, when relevant. Future research could investigate this effect further.

Our study also showed a number of non-significant effects. First, the use of the ST App did not lead to a clearer or increased shared understanding of parents’ need for care. Results show nurses more often than not succeeded in eliciting parents’ care need, which is contradicting previous studies findings [9]. Apparently, for the nurses in the current study, obtaining a shared understanding of parents’ need for care was less of a challenge. Second, when using the app, the nurses’ advice was not more usable for the parents, nor were the nurses better capable of informing the parents. This shows that there is room for improvement when it comes to the content of the app, in regard to fact sheets, videos and websites. During the study, we strived to fill the app with relevant information, but found that this was quite laborious. This may also explain why parents did not have a stronger intention to follow the advice and often opted to cope with their family issue themselves. Third, we did not find a significant effect of the app on the referral of parents. Referral was only relevant in a small number of the parents. We presumably did not have enough power to find significant differences through the surveys. However, the dossier audit did show significant differences between the two groups (i.e., fewer parents in the intervention group needed referral).

### Implications

#### Mitigating challenges in a family centred care approach

Family-centred care is increasingly being adopted in PHC and is positively valued by families and nurses [31]. Previous research has shown that family-centred practices and particularly participatory help giving have a positive influence on parenting confidence, competence, and enjoyment, which in turn have positive effects on parenting behaviour [5]. As the ST program takes a family-centred approach to PHC and has shown to facilitate participatory help giving, it can be an effective way to improve developmental and health outcomes of children [32]. By combining different functions, the use of the app mitigated the challenges defined in earlier research [8, 9]. Through textual and visual conversational support, parents were able to verbalize and prioritize their needs. After the home visit, the parents felt more empowered and better equipped to resolve family issues. These results indicate that mHealth apps can be an effective means to contribute to existing family-centred approaches.

#### Early identification of parents and their children’s’ needs

Parents are often faced with a multitude of daily family issues affecting the child’s development, such as parenting, relationships, and language. For these parents, it is not always possible to exactly pinpoint what lies at the root of these issues, and more importantly, to focus on a situation in which the problem is resolved by themselves. Once their questions or care needs are identified, simply offering generic information and education is insufficient to help these parents to cope autonomously. Many parents currently go online in search of information, but find it difficult to decide which information is most relevant and reliable for them. Sitting together with the nurse and going through the different functionalities of the app appeared to be helpful. Together they could summarize the family’s situation, set goals, and pick relevant educational materials and information to help families to achieve them.

Our expectation is that by simply providing stand-alone apps to the parent or the professional, individually, outside of the context of the home visit, this effect could not have been achieved. Through blended care, risks of crisis and issues in communication (at a distance) are avoided, and social support is facilitated [33]. Therefore, we strongly advise a similar approach for future application of (preventive) child care apps and mHealth in general.

#### Costs and benefits of the ST app

The scope of this study was establishing the effect of the ST App on the PCH, in the context of home visits. We did not study the effect of the app on the extent to which families were successful in resolving their family issues and consequently social-emotional and behavioural problems in children. Considering the current findings and the existing literature, showing the positive effects of effectiveness of home visiting programs on child outcomes [6, 7], the app may have a positive effect. However, this still needs to be validated through a study on the costs and benefits of the ST App, in terms of health care time (e.g., number of home visits and contacts between families and PCH), costs of the app, effectiveness in resolving family issues and children’s development, health and quality of life. Such a cost-benefit analysis (CBA) is currently conducted in the Netherlands [34].

#### Future use and implementation of the ST app

The results of the study provide important pointers for future adoption and implementation of apps in the context of PCH. One suggestion for improvement is to support nurses to develop the habit of using the Tablet PC and the app during home visits, by experiencing the benefits of the app themselves. Training-on-the-job could be a useful strategy to achieve this [35]. Another remark that the nurses made, is that they would have liked to easily edit and maintain the content of the app themselves. For example, an online tool for nurses to add and update their preferred folders, websites, videos and information on social services, could be added. A final suggestion for improvement was to involve the management, in an early phase of implementation, to create support for the app within the organisation. Therefore, we suggest to organize meetings to introduce the app and ask managers to facilitate their nurses to go on home visits with the app. Later on, management should be informed about the results of the use of the app.

### Limitations

First, it was beyond the scope of this study to investigate how the use of the app influenced the parents’ action after the home visit. Surveys asked the parents and nurses to evaluate the home visits, and the parent’s intention to work at their family issues and follow-up the referral. It was too burdensome for nurses to contact families to collect data on parents’ activities undertaken after the visit (in addition to the training, completing and administering surveys, and group meetings). For future studies, we strongly advise to look at the app’s effect on the parents’ activities in a follow-up.

Second, the study data has a nested structure. In both the STA and CAU group, nurses visited multiple families, completed a survey about this visit and invited parents to do the same. It may be the case that groups of parents visited by one nurse could show more similarity than responses of individual parents between nurses. In case of such as structure, an approach such as hierarchical linear modelling is favoured. However, due to privacy reasons, data were collected anonymously and we do not know which nurse saw which parents which makes such as approach impossible.

Third, the study was conducted in the city of Amsterdam (population approximately 800.000, of which almost half is immigrant). As a result, it is uncertain if our findings are replicable in rural areas. After the current study, the ST App has been piloted in two rural areas in the Netherlands. The first reactions from the nurses in these areas indicate that the app is equally beneficial. However, an important requirement is that the education and information on community services are local.

Finally, it was not possible to connect the survey and audit data. Due to privacy reasons, the audit data could not be allocated to individual families. As a result, our findings only apply to the control and intervention groups as a whole. We can state that the group with nurses using the ST App refers less to social services than the group without the app. We do not know how these findings relate to the individual characteristics of the nurses and how they used the app.

## Conclusions

The ST App contributes to the positive experience with the PCH home visits and improves communication between nurses and parents. Also, results suggest that application of the ST App in home visits improves parents’ knowledge and skills to cope with their family issues, and reduces rates of potentially redundant care. Especially for home visits to parents with a low educational level, the app has an additional value. Overall, it can be concluded that applying mobile applications, such as the ST App, can contribute to the quality of care within family centred care approaches in PCH and increase parent empowerment.

## Abbreviations

CAU: Control group applying Care As Usual
PCH: Preventive child health care
SFBT: Solution focused brief therapy
ST App: StartingTogether App
ST: StartingTogether
STA: Intervention group using StartingTogether App
